# Influence of tumour size on human prostate tumour metastasis in athymic nude mice.

**DOI:** 10.1038/bjc.1985.57

**Published:** 1985-03

**Authors:** J. L. Ware, E. R. DeLong


*Br. J. Cancer* (1985), **51**, 419–423

### Short Communication

### Influence of tumour size on human prostate tumour metastasis in athymic nude mice

J.L. Ware[1] & E.R. DeLong[2]

[1]*Department of Surgery, Division of Urology and* [2]*Department of Community and Family Medicine, Division of Biometry, Duke University Medical Center, Durham, NC 27710 USA.*

Human tumours which grow and metastasize in immunodeficient animals are amenable to controlled investigation of human tumour metastasis in the presence of the original tumour inoculum. In previous reports (Ware *et al.*, 1982, 1984), we described a unique human prostate carcinoma subline, 1-LN-PC-3-1A, which consistently metastasizes from a subcutaneous (s.c.) site to both regional and distant lymph nodes in tumour-excised adult athymic nude mice. These tumour cells also form multiple lung micrometastases in 30–50% of tumour-excised mice (Ware *et al.*, 1984).

We have used this model system to address two basic questions concerning spontaneous metastasis of the human prostate carcinoma subline 1-LN-PC-3-1A growing subcutaneously in adult nude mice: (i) What are the characteristics of metastasis by these cells in the presence of the original s.c. tumour, and (ii) Is the size of the s.c. tumour a significant determinant of lymphatic metastasis in this experimental system?

Male athymic nude mice (nu/nu) (BALB/cAnBOM), 6–8 weeks old, were used in all experiments. Specific pathogen-free mice were obtained from two sources. Groups 1 and 2 were purchased from Harlan-Sprague Dawley (Indianapolis, Indiana). These mice were received from the supplier at the age of 4–5 weeks and allowed to acclimatize for 2 more weeks in a barrier facility prior to any experimentation. Groups 3 and 4 were obtained from the breeding colony of athymic nude mice maintained by the Urology Research Laboratories, Duke University Medical Center. All of these mice (groups 1, 2, 3 and 4) were provided with sterile food, water, bedding and cages, and maintained in an isolated barrier facility with strict access limitations. Heterozygous (nu/+) mice were maintained under the same conditions as the experimental animals to act as additional monitors for microbial contamination. No evidence of nonspecific bacterial contamination or parasitic infection was found for mice involved in these experiments. Furthermore, sera from the heterozygous sentinal mice were screened by the Veterinary Diagnostic Laboratory, University of Missouri School of Veterinary Medicine (Columbus, Missouri) and found to be free of significant titres of antibody to any of the 11 most common murine viruses, including murine hepatitis virus.

The utilization of mice from 2 different suppliers served two functions. It allowed inclusion of sufficient numbers of nude mice in each group to permit meaningful statistical analysis and it also ensured that the results obtained would not be unique to mice from a single source. The mice were divided into 4 chronologically spaced groups, 2 from each supplier, in an effort to eliminate time and batch biases and thus to allow identification of effects which would be general across time.

Human prostate carcinoma cells designated 1-LN-PC-3-1A were derived from a spontaneous lymph node metastasis originating in a nude mouse bearing a PC-3-1A tumor as described previously (Ware *et al.*, 1982). PC 3-1A (Ware *et al.*, 1982) was obtained from the established human prostate carcinoma cell line PC-3 (Kaighn *et al.*, 1979). Cells used in these experiments were recovered from frozen stocks stored in liquid nitrogen and maintained *in vitro* no more than 3 weeks prior to injection. These cells were grown in RPMI 1640 supplemented with 10% heat-inactivated calf serum (HYCLONE Laboratories, Logan, Utah) and gentimicin ($50 \mathrm{mg\,l^{-1}}$). All cells were free of mycoplasma contamination as revealed by cytoplasmic staining with quiniquine dihydrochloride. Cells were collected by gentle scraping with a sterile rubber policeman and were $\geq 95\%$ viable by trypan blue exclusion.

Groups of mice were injected s.c. with $10^6$ 1-LN cells in a volume of 0.2 ml PBS on the lower left dorsal surface. Mice were randomized to be sacrificed 21, 26, 31 or 36 days after the initial s.c. injection. Tumours grew s.c. in 100% of the injected animals and were measurable 9 days after

Correspondence: J.L. Ware, Box 3062, Duke Univ. Medical Center, Durham NC 27710 USA.
Received 21 June 1984; and in revised form 8 November 1984.

© The Macmillan Press Ltd., 1985

injection. Growth of the s.c. tumour was monitored 3 times a week by measurement with vernier calipers. The volume of the s.c. tumour was calculated by the following formula:

$$V = \frac{l \times w^2}{2}$$

where $l$ = length and $w$ = width. This geometric formula for approximate calculation of the volume of an ellipsoid is commonly used to estimate s.c. tumour volume from linear dimensions with reasonable accuracy (Steele, 1977; Norton & Simon, 1979). The tumours described in this study appeared to grow as prolate spheroids during the exponential phase of growth and the tumour volumes used in this analysis were measured during that phase.

The average s.c. tumour volume doubling time was 3.5 days. On the day of sacrifice, the s.c. tumour was removed and weighed. The site of the original tumour inoculum, designated "primary", was examined for gross evidence of invasion of muscle or the peritoneal membrane. At autopsy the left and right inguinal lymph nodes, the axillary lymph nodes, and the brachial lymph nodes were removed and preserved in 10% buffered formalin. Any grossly enlarged internal lymph nodes, as well as the lungs, were also removed and preserved for histological examination.

Lymph nodes were embedded in paraffin and semi-serially sectioned (5 $\mu$m thickness) at 80 $\mu$m intervals. Lungs were subjected to routine sectioning procedures which had previously disclosed multiple lung micrometastases in tumour-excised mice (Ware *et al.*, 1984). All tissues were stained with hematoxylin and eosin. Micrometastases were defined as a focus of 20 or more tumour cells detected in a 5 $\mu$m section. Lymph nodes removed from mice later than 21 days after injection often contained hundreds of tumour cells which displaced half of the normal lymphoid architecture.

In order to determine which variables significantly influenced development of micrometastases, logistic regression analyses were performed. This type of analysis models the probability of response (in this case, development of histologically detectable metastases) as a logistic function of the variables considered. This approach is commonly used in bioassay experiments (Finney, 1978). To evaluate whether other effects were being confounded by sacrifice time, we performed all analyses with and without controlling for time of sacrifice. Linear contrasts were used to detect effects due to different sacrifice times (Steel & Torrie, 1980).

The 1-LN-PC-3-1A cells consistently metastasized to the superficial lymph nodes of adult athymic nude mice in the presence of the original s.c. tumour. Both regional and distant lymphatic metastases were observed among these mice. Internal lymph node metastases were also found in mice sacrificed at 31 or 36 days. Furthermore, internal lymphatic metastases were present in some mice which were free of superficial lymphatic metastases. Three mice sacrificed at day 31 and two mice sacrificed at 36 days had enlarged renal and/or paraaortic lymph nodes with histologically confirmed metastases. However, all superficial lymph nodes, including the ipsilateral inguinal lymph node, were free of micrometastases. Lung micrometastases were rarely found during routine sectioning (1/8 mice sacrificed 36 days after s.c. injection). Only 4/58 mice examined had s.c. tumours which were macroscopically invasive at autopsy.

As indicated in Table I, for the combined data from groups 1, 2, 3 and 4, 11/15 (73%) mice sacrificed 21 days after injection had 1 or more lymphatic metastases, while 9/18 (50%) mice had micrometastases at day 26. Among mice sacrificed 31 and 36 days after injection, metastases were detected in 12/17 (71%) and 7/8 (88%) respectively. The logistic regression analysis indicated no significant difference in probability of metastases due to time of sacrifice ($P$=0.27). However, in a preliminary analysis of groups 1, 2 and 3, a pronounced reduction in incidence of metastases was noted at day 26. Linear contrasts on this effect pinpointed a barely significant ($P$=0.05) decrease in percentage of mice with metastases between days 21 (75%) and 26 (36%), followed by an increase ($P$=0.06) from day 26 to day 31 (71%). The mean number of lymph node metastases per mouse was

**Table I** Proportion of tumour-bearing mice with lymphatic metastases at different times after injection

| | *No. of mice with lymphatic metastases/ no. of mice sacrificed at the indicated day after injection* | | | |
|---|---|---|---|---|
| *Group* | *21* | *26* | *31* | *36* |
| 1 | 2/3 | 2/5 | 4/5 | 3/3 |
| 2 | 3/3 | 2/5 | 2/5 | 3/3 |
| 3 | 4/6 | 1/4 | 4/4 | 1/2 |
| 4 | 2/3 | 4/4 | 2/3 | ND |
| Total (% All) | 11/15 (73%) | 9/18 (50%) | 12/17 (71%) | 7/8 (88%) |

ND = Not Done.

1.3, 1.0, 1.5 and 1.4 for mice sacrificed on days 21, 26, 31, and 36 respectively.

Macroscopically visible lymphatic metastases were not observed until 26 days after injection in any mice. The development of macrometastases increased in parallel to the increase in micrometastases from days 26 to 36 (Figure 1).

**Figure 1** Comparison of percentage of nude mice with histologically detected lymphatic micrometastases (○) with percentage of nude mice also having macrometastases (●) of the lymph nodes. (Combined data. groups 1, 2, 3 and 4. See **Table I**).

In general, the size of s.c. tumour correlated positively with the probability of lymphatic metastasis, i.e., the larger the tumour, the greater the likelihood of lymphatic metastasis. The most influential variable in our analyses was tumour volume 20 days after s.c. injection (VOL20). Regardless of which combinations of variables were modeled, VOL20 consistently emerged as a statistically significant predictor of tumour metastases, with higher volume yielding higher probabilities of metastases (Table II). Tested alone in the model using the combined data from groups 1, 2, 3, and 4, VOL20 had a $P$-value of 0.004; after controlling for day of sacrifice, the $P$-value remained at 0.006. Furthermore, the effect was consistent over the four sacrifice dates (i.e., there was not a statistically significant, nor visually evident, interaction effect between this variable and day of sacrifice). Volume measured on Day 9 also correlated positively with the probability of metastasis, but was less significant statistically ($P=0.07$). The greater significance of s.c. volume calculated at Day 20 may have reflected greater accuracy in caliper measurements. Nine days post-injection was the earliest date at which the s.c. tumours were measurable, and accurate length and width measurements were more difficult to obtain at Day 9, thus contributing to greater potential error in volume calculations.

The weight of the s.c. tumour at the time of sacrifice also had a positive influence on development of metastases ($P=0.03$ alone and $P=0.02$ after controlling for sacrifice time) (Table III). This result is consistent with the finding for volume at day 20, since tumour weight and volume are highly correlated. In fact, among those mice sacrificed at Day 21, the correlation between volume at Day 20 and sacrifice weight was 0.85 ($P=0.0001$).

The relation between specific growth parameters of a primary tumour and its metastases may be positive, negative, or nonexistent, depending upon the host/tumour system examined. The metastatic behaviour of several animal tumours has been shown to be influenced by the presence or absence (Gorelik, 1983), size (Anderson *et al.*, 1974), and/or growth rate (Dewys, 1972) of the primary tumour.

**Table II** Effect of s.c. tumour volume on lymphatic metastases in tumour-bearing mice

| | *Groups 1, 2, 3 and 4* | | | |
|---|---|---|---|---|
| | *Day of sacrifice* | | | |
| | *21* | *26* | *31* | *36* |
| Incidence of metastases (from **Table I**) | 11/15 | 9/18 | 12/17 | 7/8 |
| Mean volume Day 20 (±s.d.) ($mm^3$) for: | | | | |
| Mice without metastases | 212 (±51) | 253 (±86) | 229 (±117) | 224[a] |
| Mice with metastases | 387 (±172) | 348 (±195) | 366 (±111) | 300 (±85) |

Significance of volume at Day 20 as a predictor of lymphatic metastasis: $P=0.004$.
[a]Indicates only one mouse in this category, therefore no s.d. possible.

**Table III** Effect of s.c. tumour weight at date of sacrifice on lymphatic metastases in tumour-bearing mice

| | *Groups 1, 2, 3 and 4* *Day of sacrifice* | | | |
|---|---|---|---|---|
| | *21* | *26* | *31* | *36* |
| Incidence of metastases (from **Table I**) | 11/15 | 9/18 | 12/17 | 7/8 |
| Weight (g) (±s.d.): | | | | |
| Mice without metastases | 0.220 (±0.08) | 0.710 (±0.29) | 0.570 (±0.23) | 1.11[a] |
| Mice with metastases | 0.390 (±0.19) | 0.770 (±0.40) | 1.47 (±0.65) | 1.54 (±0.87) |

Significance of s.c. tumour weight at day of sacrifice as a predictor of lymphatic metastases: $P = 0.03$.
[a]Indicates only one mouse in this category, therefore no s.d. possible.

In other cases, no correlation between growth rate (Hager *et al.*, 1978) or primary tumour size (Price *et al.*, 1982) and metastatic incidence has been observed. The influence of the size of a *human* tumour on metastatic behaviour in immunodeficient hosts is relatively unexplored. The reproducible lymphatic metastases by 1-LN-PC-3-1A cells in tumour-bearing nude mice provided the opportunity to analyze the relation between s.c. tumour size and the probability of metastases for this human prostate tumour. Furthermore, this study demonstrated the important role of statistical methods in the design and analysis of this type of biological experiment.

Using logistic regression analyses, we demonstrated that the size of the s.c. tumour correlated positively with the probability of lymphatic metastasis among these 58 nude mice. The most influential variable was the calculated s.c. tumour volume 20 days after inoculation. After controlling for time (day of sacrifice), VOL20 was still a significant factor. Thus s.c. tumour *size*, rather than duration of growth, appeared to be the important parameter. Furthermore, the weight of the s.c. tumour at the day of sacrifice also had a positive influence on development of metastases. Thus the findings for two different expressions of tumour size were consistent with each other.

We believe that our randomized design enhances the significance of these results. Immediately after tumour inoculation, each mouse received a randomly generated sacrifice time (21, 26, 31, or 36 days). Thus no subconscious bias could have led us to consistently sacrifice mice with certain attributes, e.g., large tumours, either early or late in the schedule.

We also believe that our statistical analysis is the appropriate one for this study design. Rather than comparing results obtained at individual sacrifice days separately, we have incorporated data for all sacrifice days into a unified analysis. We have thus enhanced the power of analysis while decreasing the number of statistical tests performed.

The mean number of metastases per mouse was relatively constant, 1–2 per mouse. This contrasts with previous investigations of the 1-LN-PC-3-1A line in tumour-excised nude mice. Among mice sacrificed 4 weeks after tumour excision (8 weeks post-injection), multiple lymph node metastases were often found per mouse and lung micrometastases were demonstrable in half the mice in routine lung sections (Ware *et al.*, 1984; Ware, unpublished observations). These experiments are not strictly comparable to the ones described in this report due to temporal differences. Nonetheless, the rarity of pulmonary micrometastases and the restriction of the number of lymphatic metastases per mouse among tumour-bearing mice were striking. This apparent difference in metastatic dissemination between tumor-excised and tumour-bearing mice has been observed by other investigators working with human tumours grown in nude mice. The excision of several human melanomas (Wilson *et al.*, 1980), human breast carcinomas (Ozzello & Sordat, 1980), and human colon carcinoma cells (Sordat *et al.*, 1982) growing s.c. is reported to promote or permit greater dissemination of these tumours in adult nude mice.

As a variation on approaches taken by other investigators, we chose to begin analyzing the relation between the size of a human tumour and its metastases in nude mice without disturbing (i.e., excising) the s.c. tumour. Application of statistical methodology in both experimental design and interpretation of the data permitted identification of a positive correlation between s.c. tumour size and the probability of lymphatic metastasis by the human prostate carcinoma cells, 1-LN-PC-3-1A. This finding provides a foundation for future experimental analysis of the mechanisms underlying this size-metastasis relation for a human tumour growing in a nude mouse.

This research was supported by NCI grant CA33208, Comprehensive Cancer Center Core Grant CA-00726, and the CURED Foundation.

### References

ANDERSON, J.C., FUGMANN, R.A., STOLFI, R.L. & MARTIN, D.S. (1974). Metastatic incidence of a spontaneous murine mammary adenocarcinoma. *Cancer Res.*, **34,** 1916.

DEWYS, W.D. (1972). Studies correlating the growth rate of a tumor and its metastases and providing evidence for tumor-related systemic growth-retarding factors. *Cancer Res.*, **32,** 374.

FINNEY, D.J. (1978). *Statistical Methods in Biological Assay.* 3rd Ed. London: Charles Griffin & Co.

GORELIK, E., (1983). Resistance of tumor-bearing mice to a second tumor challenge. *Cancer Res.*, **43,** 138.

HAGER, J.C., MILLER, R.F. & HEPPNER, G.H. (1978). Influence of serial transplantation on the immunological and clinical correlates of BALB/cFC3H mouse mammary tumors. *Cancer Res.*, **38,** 2492.

KAIGHN, M.E., NARAGAN, K.S., OHMUKI, Y., LECHNER, J.F. & JONES, L.W. (1979). Establishment and characterization of a human prostatic carcinoma cell line (PC-3). *Invest. Urol.*, **17,** 16.

NORTON, L. & SIMON, R. (1979). New thoughts on the relationship of tumor growth characteristics to sensitivity to treatment. In: *Methods in Cancer Res.* (Eds. DeVita & Busch), New York: Academic Press, XVII, p. 53.

OZZELLO, I.L. & SORDAT, M. (1980). Behavior of tumors produced by transplantation of human mammary cell lines in athymic nude mice. *Eur. J. Cancer*, **16,** 553.

PRICE, J.E., CARR, D., JONES, L.D., MESSER, P. & TARIN, D. (1982). Experimental analysis of factors affecting metastatic spread using naturally occuring tumors. *Invasion Metast.*, **2,** 77.

SORDAT, B., UEYAMA, Y. & FOGH, J. (1982). Metastasis of tumor xenografts in the nude mouse. In *The Nude Mouse in Experimental and Clinical Research.* (Eds. Fogh & Govianella), New York: Academic Press, Vol. 2, p. 95.

STEELE, G.G. (1977). *Growth Kinetics of Tumours.* Oxford: University Press.

STEEL, R.G.D. & TORRIE, J.H. (1980). *Principles and Procedures of Statistics: A Biometrical Approach.* 2nd Ed. New York: McGraw-Hill.

WARE, J.L., PAULSON, D.F., MICKEY, G.H. & WEBB, K.S. (1982). Spontaneous metastasis of cells of the human prostate carcinoma cell line PC-3 in athymic nude mice. *J. Urol.*, **128,** 1064.

WARE, J.L., PAULSON, D.F., VOLLMER, R.T. & WEBB, K.S. (1984). Cellular phenotype and spontaneous metastasis of human prostate carcinoma cells (PC-3) in the athymic nude mouse. In: *Immune-Deficient Animals. 4th Int. Workshop on Immune Deficient Animals in Experimental Research.* Basel: Karger, p. 345.

WILSON, E.L., GARTNER, M., CAMPBELL, J.A.H. & DOWDLE, E.B. (1984). Growth and behavior of human melanomas in nude mice: Effect of fibroblasts. In: *Immune-Deficient Animals. 4th Int. Workshop on Immune-Deficient Animals in Experimental Research.* (Ed. Sordat), Basel: Karger, p. 357.


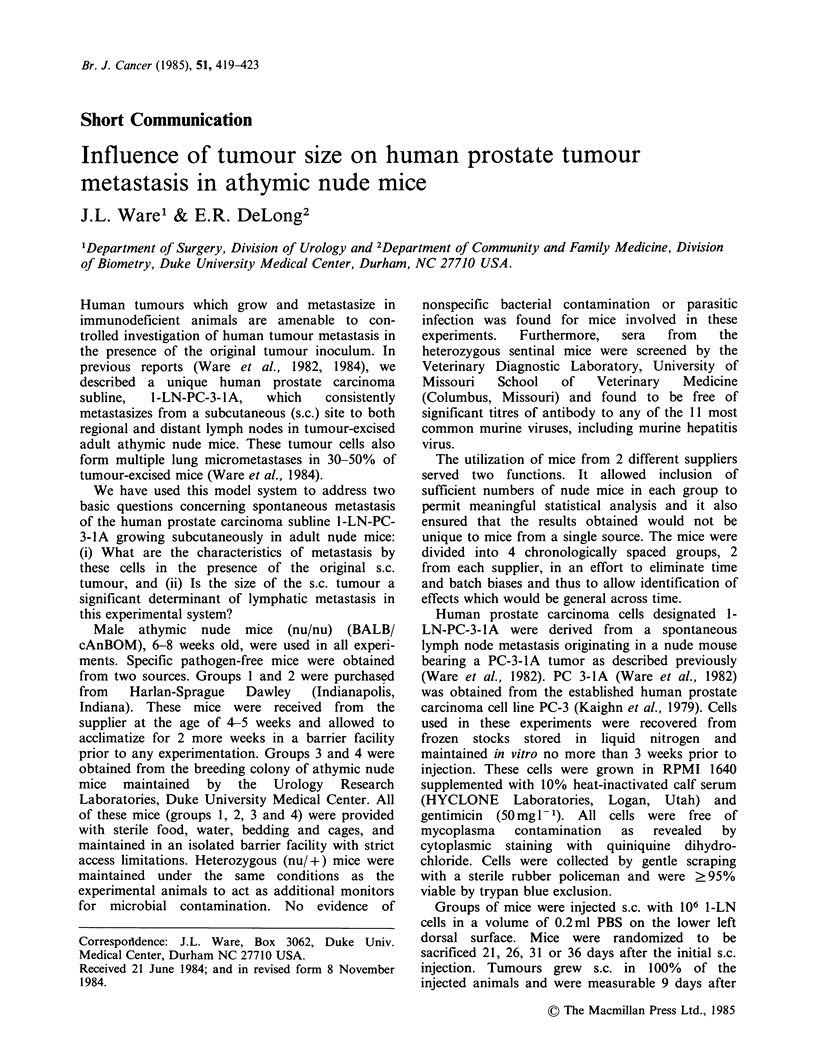

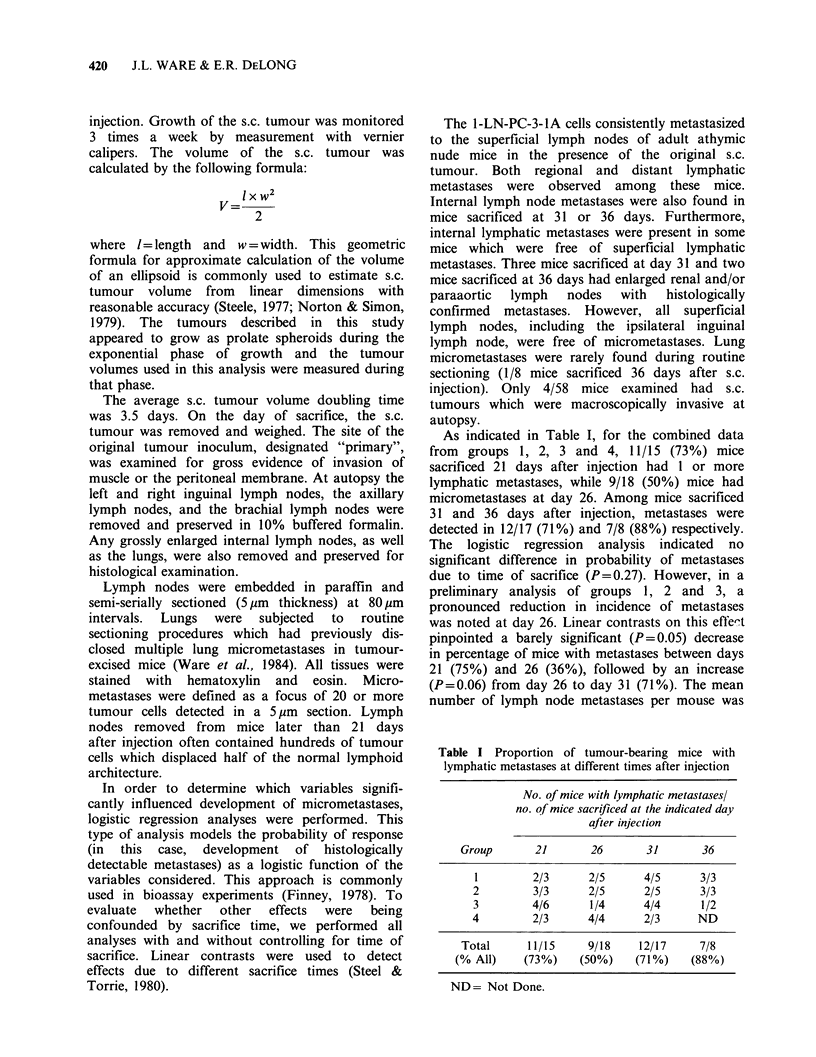

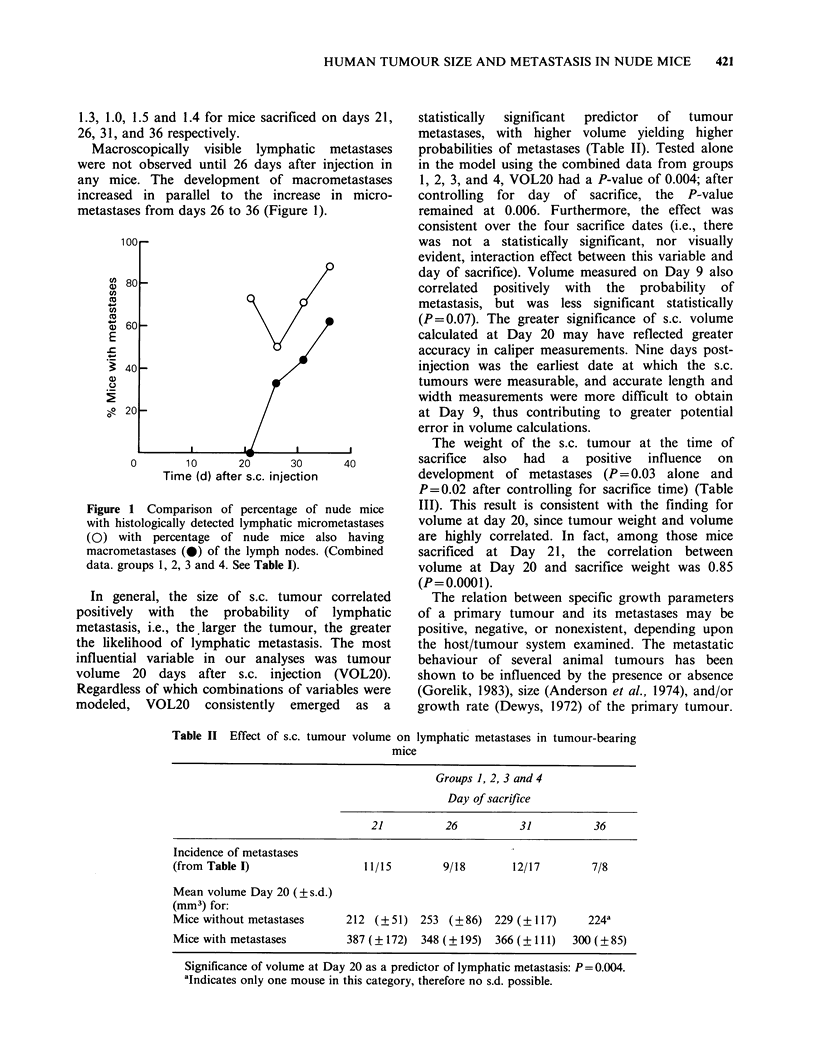

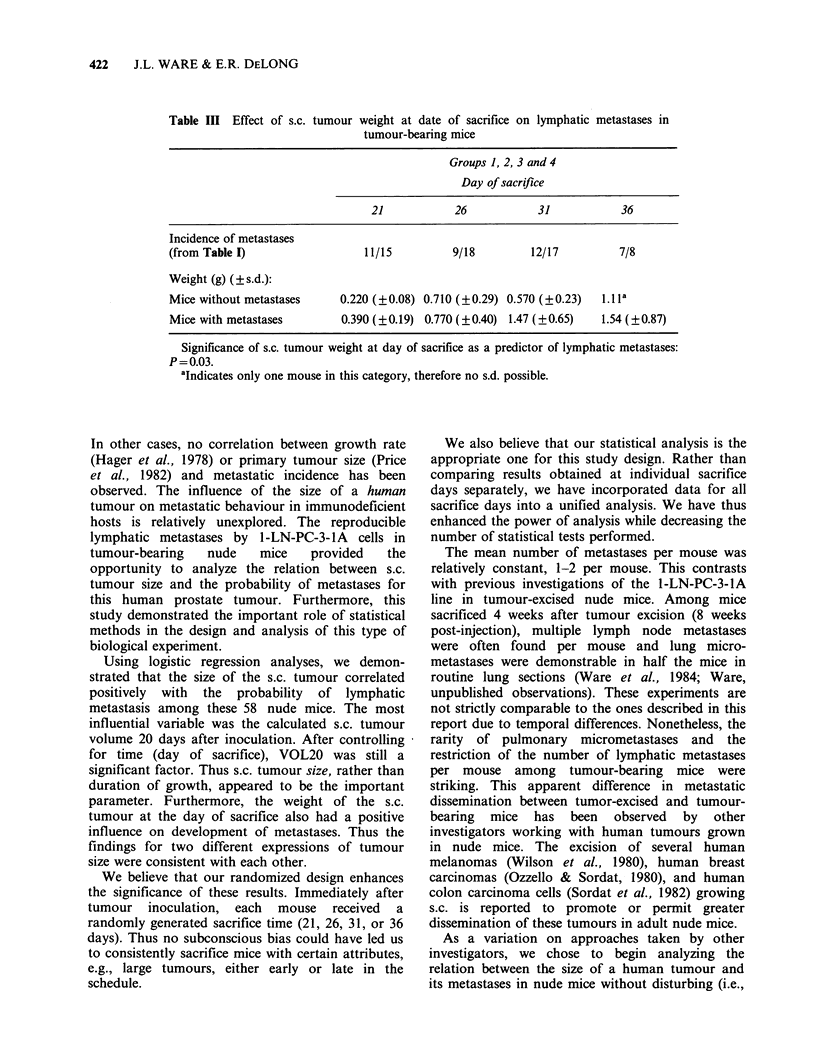

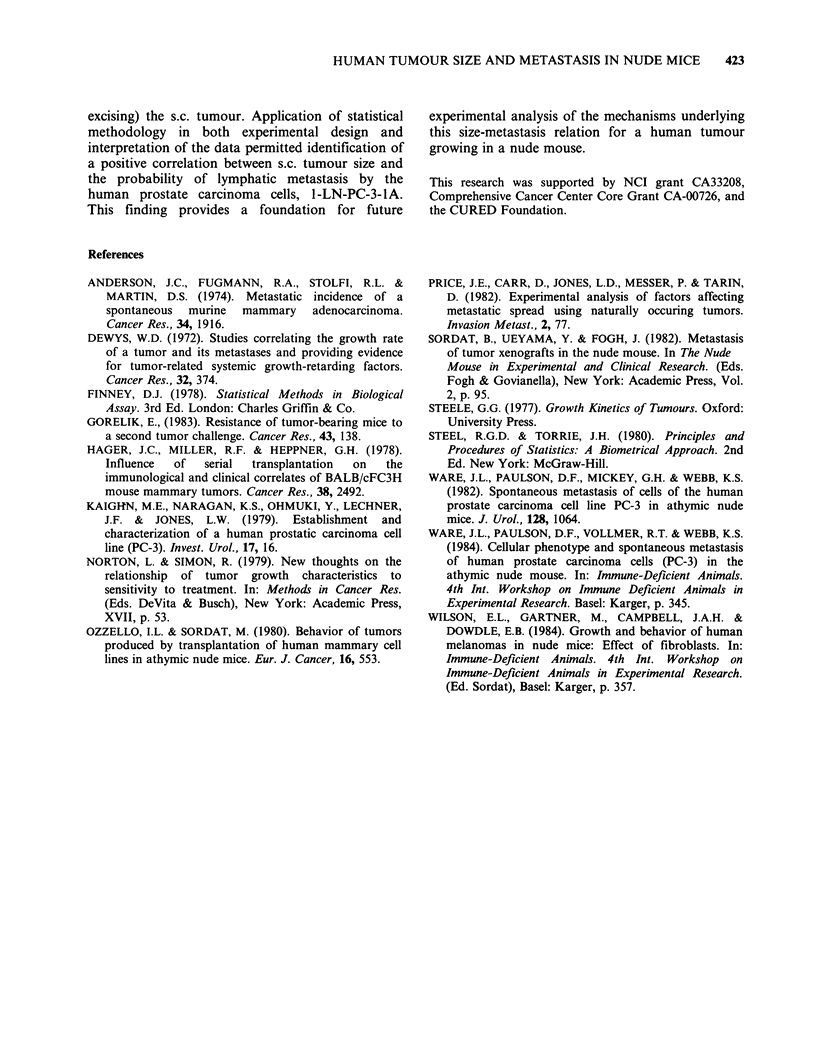


## References

[OCR_00483] Anderson J. C., Fugmann R. A., Stolfi R. L., Martin D. S. (1974). Metastatic incidence of a spontaneous murine mammary adenocarcinoma.. Cancer Res.

[OCR_00489] DeWys W. D. (1972). Studies correlating the growth rate of a tumor and its metastases and providing evidence for tumor-related systemic growth-retarding factors.. Cancer Res.

[OCR_00499] Gorelik E. (1983). Resistance of tumor-bearing mice to a second tumor challenge.. Cancer Res.

[OCR_00503] Hager J. C., Miller F. R., Heppner G. H. (1978). Influence of serial transplantation on the immunological-clinical correlates of BALB/cfC3H mouse mammary tumors.. Cancer Res.

[OCR_00509] Kaighn M. E., Narayan K. S., Ohnuki Y., Lechner J. F., Jones L. W. (1979). Establishment and characterization of a human prostatic carcinoma cell line (PC-3).. Invest Urol.

[OCR_00522] Ozzello L., Sordat M. (1980). Behavior of tumors produced by transplantation of human mammary cell lines in athymic nude mice.. Eur J Cancer.

[OCR_00527] Price J. E., Carr D., Jones L. D., Messer P., Tarin D. (1982). Experimental analysis of factors affecting metastatic spread using naturally occurring tumours.. Invasion Metastasis.

[OCR_00550] Ware J. L., Paulson D. F., Mickey G. H., Webb K. S. (1982). Spontaneous metastasis of cells of the human prostate carcinoma cell line PC-3 in athymic nude mice.. J Urol.

